# Supplementary material for: Mining and identification of polyunsaturated fatty acid synthesis genes active during camelina seed development using 454 pyrosequencing
Source: BMC Plant Biol. 2015 Jun 18;15:147. doi: 10.1186/s12870-015-0513-6 (PMC4470060; doi:10.1186/s12870-015-0513-6)
Supplement: Additional file 1: Fig. S2. — The quality analysis of mRNA and cDNA from C. sativa seeds. The mRNA and cDNA were examined by electrophoresis and shown in (A) and (B). The qualities of mRNA for the construction of cDNA library were further analyzed by Agilent2100 (C-F). [file 12870_2015_513_MOESM1_ESM.png]

Mining and identification of polyunsaturated fatty acid synthesis genes active during camelina seed development using 454 pyrosequencing

Fawei Wang[1], Huan Chen[2], Xiaowei Li[1], Nan Wang[1], Tianyi Wang[2], Jing Yang[1], Lili Guan[1], Na Yao[1], Linna Du[1], Yanfang Wang[1], Xiuming Liu[1], Xifeng Chen[3], Zhenmin Wang[3], Yuanyuan Dong[1*], Haiyan Li[1,2*].

Affiliations

[1]Ministry of Education Engineering Research Center of Bioreactor and Pharmaceutical Development, Jilin Agricultural University, Changchun, Jilin 130118, China. [2]College of life Sciences, Jilin Agricultural University, Changchun, Jilin 130118, China. [3]Jilin Technology Innovation Center for Soybean Region, Jilin Agricultural University, Changchun, Jilin 130118, China.

Fawei Wang: E-mail: fw-1980@163.com

Huan Chen: E-mail: chjlau@163.com

Xiaowei Li: E-mail: xiaoweili1206@163.com

Nan Wang: E-mail: wangnanlunwen@126.com

Jing Yang: E-mail: yangjing5122010@163.com

Lili Guan: E-mail: guanll2004@163.com

Na Yao: E-mail: 52730603@qq.com

Linna Du: E-mail: dulinna0918@163.com

Yanfang Wang: E-mail: nifengcao_2000@163.com

Xiuming Liu: E-mail: xiuming1211@163.com

Xifeng Chen: E-mail: chenxifeng2003@126.com

Zhenmin Wang: E-mail: wangzhenmin2004@sina.com

Yuanyuan Dong: E-mail: dongyuanyuan_dyy@yahoo.com.cn

Haiyan Li: E-mail: hyli99@163.com

*Corresponding authors.

Abstract

Background: Camelina (*Camelina sativa L.*) is well known for its high unsaturated fatty acid content and great resistance to environmental stress. However, little is known about the molecular mechanisms of unsaturated fatty acid biosynthesis in this annual oilseed crop. To gain greater insight into this mechanism, the transcriptome profiles of seeds at different developmental stages were analyzed by 454 pyrosequencing.

Results: Sequencing of two normalized 454 libraries produced 831,632 clean reads. A total of 32,759 unigenes with an average length of 642 bp were obtained by *de novo* assembly, and 12,476 up-regulated and 12,390 down-regulated unigenes were identified in the 20 DAF (days after flowering) library compared with the 10 DAF library. Functional annotations showed that 220 genes annotated as fatty acid biosynthesis genes were up-regulated in 20 DAF sample. Among them, 47 candidate unigenes were characterized as responsible for polyunsaturated fatty acid synthesis. To verify unigene expression levels calculated from the transcriptome analysis results, quantitative real-time PCR was performed on 11 randomly selected genes from the 220 up-regulated genes; 10 showed consistency between qRT-PCR and 454 pyrosequencing results.

Conclusions: Investigation of gene expression levels revealed 32,759 genes involved in seed development, many of which showed significant changes in the 20 DAF sample compared with the 10 DAF sample. Our 454 pyrosequencing data for the camelina transcriptome provide an insight into the molecular mechanisms and regulatory pathways of polyunsaturated fatty acid biosynthesis in camelina. The genes characterized in our research will provide candidate genes for the genetic modification of crops.

Keywords: *Camelina sativa*, Oil crop, Polyunsaturated fatty acid, Transcriptome, Gene

expression, qRT-PCR

Background

Polyunsaturated fatty acids (PUFAs) are fatty acids that contain more than one double bond in their backbone. They include many important compounds such as essential fatty acids (omega-3 and omega-6 fatty acids) that human beings and animals cannot synthesize and need to acquire through food. Fish oil and vegetable oil supplements are the main sources of PUFAs. Vegetable oils, such as soybean oil, contain about 7% alpha-linolenic acid (ALA) (omega-3 fatty acid) and 52% linoleic acid (LA) (omega-6 fatty acid) [1]. The optimal dietary fatty acid profile includes a low intake of both saturated and omega-6 fatty acids and a moderate intake of omega-3 fatty acids [2]. However, the majority of vegetable oils contains excessive amounts of omega-6 fatty acids but are deficient in omega-3 fatty acids, except for camelina oil and linseed oil. Modulation of omega-3/omega-6 polyunsaturated fatty acid ratios has important implications for human health.

Camelina sativa is a flowering plant in the family Brassicaceae and is usually known as camelina. This plant is cultivated as an oilseed crop mainly in Europe and North America. The dominant fatty acids of camelina oil are omega-3 fatty acid (31.1%) and omega-6 fatty acid (25.9%) [3]. Importantly, camelina oil also contains high levels of gamma-tocopherol (vitamin E), which protects against lipid oxidation [4]. The fatty acid composition of camelina oil is especially suitable for human health. However, the mechanisms of polyunsaturated fatty acid synthesis in *C. sativa* are still unknown. In recent years, researchers have paid more and more attention to camelina. Hutcheon et al. [5] characterized two genes of the fatty acid biosynthesis pathway, fatty acid desaturase (FAD) 2 and fatty acid elongase (FAE) 1, which revealed that *C. sativa* be

considered an allohexaploid. The allohexaploid nature of the *C. sativa* genome brings more complexity in the biosynthesis of PUFAs. Moreover, the functions of three CsFAD2 were further studied soon after [6]. Furthermore, the genome of *C. sativa* has been sequenced and annotated [7]. *C. sativa* could also be used as a recipient to overexpress PUFA synthesis genes and produce more PUFAs, such as omega-3 or omega-6 fatty acids [8-10]. In previous studies, the transcriptome analysis of *C. sativa* had carried out by 454 sequencing, Illumina GAIIX sequencing and paired-end sequencing [11-13]. However, the mechanism of PUFA biosynthesis in *C. sativa* remains unclear and difficult to predict.

To comprehensively understand the molecular processes underlying the seed development of *C. sativa*, we characterized the transcriptome of seeds at different developmental stages. We generated 831,632 clean reads and obtained 32,759 unigenes from seed samples. We then matched the unigenes to 187 pathways and identified 47 PUFA biosynthesis related genes. We verified the expression levels of 11 randomly selected genes from 220 up-regulated genes, 10 of which showed the same results in both qRT-PCR and sequencing. To our knowledge, this is the first genome-wide study of transcript profiles in *C. sativa* seeds at different developmental stages. The assembled, annotated unigenes and gene expression profiles will facilitate the identification of genes involved in PUFA biosynthesis and be a useful reference for other *C. sativa* developmental studies.

Results

Lipid accumulation at different stages during seed development

To characterize the polyunsaturated fatty acid (PUFA) synthesis genes in camelina, we quantified

the lipid contents in camelina seeds harvested from 10 to 40 days after flowering (DAF). After testing, we found that the lipid content was very low in seeds at 10 DAF. The lipid contents increased dramatically during 10 to 25 DAF, reached a maximum level at 25 DAF, and then remained steady until 40 DAF (Figure 1). According to this result, 10 DAF and 20 DAF seed samples were used for transcriptome sequencing analysis to explore PUFA synthesis genes.

Sequencing output and assembly

Total RNA was extracted from the seeds of *C. sativa*. The quality of RNA and cDNA were examined by electrophoresis and Agilent2100, which were shown in Additional file 2: Fiugre S2. The cDNA libraries form 10 DAF and 20 DAF were subjected to 454 pyrosequencing. After sequencing, a total of 529,324 and 318,804 high-quality transcriptomic raw sequence reads were obtained from the 10 DAF and 20 DAF samples, respectively (Table 1). To obtain clean reads, contaminating sequences, low quality reads, short reads, highly repetitive sequences and vector sequences were filtered out. Finally, 521,507 and 310,125 clean reads were obtained from 10 DAF and 20 DAF with average lengths of 630 bp and 654 bp. Furthermore, 25,398 and 23,678 unigenes were assembled based on the clean reads of these two samples. The size distribution of these unigenes is shown in Figure 2. The longest unigene was 7,043 bp. Most of the unigenes (80.72%) were distributed in the 200–1,000 bp region, while unigenes of 1,001–2,000 bp length accounted for 9.5% of the total. Of these genes, 9,081 were unique to 10 DAF and 7,361 were unique to 20 DAF (Figure 3). The differences in unique genes were of interest because of their potential importance at each stage.

Transcriptional profile analysis of unigenes during seed development

Differentially transcribed sequences were analyzed in the 10 DAF and 20 DAF samples to

characterize the PUFA synthesis genes. Of the 32,759 total genes, 12,476 up-regulated genes (log2 ratio (20 DAF/10 DAF) ≥ 1) and 12,390 down-regulated genes (log2 ratio (10 DAF/20 DAF) ≥ 1) were predicted to be significantly differentially expressed genes (DEGs) in the 20 DAF sample compared with 10 DAF (Figure 4A). The transcriptional levels of 15.61% of unigenes increased more than 2-fold in 20 DAF and 9.64% of genes increased more than 2-fold in 10 DAF (Figure 4B). The differences in the expression of shared genes were of interest to discover PUFA synthesis genes active throughout seed development. Next, the unigenes were analyzed using the COG and KEGG pathway databases for functional annotation.

Functional annotation and classification

To identify which pathways they belonged to, the unigenes were annotated using the COG, KEGG and other databases. The number of matched proteins in different databases was summarized in the Additional file 6: Table S4. Twenty-five functional categories were identified by COG classification (Figure 5). General function proteins represented the largest category, comprising about 16.46% of all unigenes. The next largest category was the “posttranslational modification, protein turnover, chaperones” group (14.323%). “Lipid transport and metabolism”, which we focused on, comprised about 3.503%. Furthermore, gene annotation based on the DEGs was carried out. There were more up-regulated genes (log2 ratio (20 DAF/10 DAF) ≥ 1) than down-regulated genes (log2 ratio (10 DAF/20 DAF) ≥ 1) in all categories, except “cytoskeleton” (Figure 6).

In the KEGG pathway annotation, 187 pathways were matched as shown in Additional file 3: Table S1. KEGG pathway network analysis showed that there are 11 and 69 up-regulated unigenes in the “fatty acid biosynthesis” pathway in 10 DAF (10 DAF vs 20 DAF) and 20 DAF (20 DAF vs

10 DAF) samples, respectively. Many genes encoding enzymes were found in this pathway, such as acetyl-CoA carboxylase (6.4.1.2, 6.3.4.14), enoyl-acyl carrier protein reductase (FabK), 3-ketoacyl-acyl carrier protein reductase (FabG) and acyl-acyl carrier protein desaturase (1.14.192) (Figure 7). FabF, which catalyzes the condensation reaction of fatty acid synthesis by the addition of two carbons to an acyl acceptor, was down-regulated in this pathway. In addition, 51 and 98 up-regulated genes were found in 10 DAF (10 DAF vs 20 DAF) and 20 DAF (20 DAF vs 10 DAF) in the "biosynthesis of unsaturated fatty acids" pathway (Additional file 3: Table S1). However, the only one gene encoding acyl-CoA thioesterase (3.1.2.2) was matched to 22 reactions (Additional file 1: Figure S1).

DEGs related to PUFA biosynthesis

After gene functional annotation, we searched for fatty acid synthesis genes among the unigenes. We found 220 up-regulated fatty acid biosynthesis genes in the 20 DAF sample (Additional file 4: Table S2). In this group, 47 PUFA synthesis related genes were discovered (Table 2). Most of them were annotated as omega 6 fatty acid desaturase (10 genes), delta-9 acyl-lipid desaturase (8 genes) and long chain acyl-CoA synthetase (7 genes). Omega 6 fatty acid desaturase and delta-9 acyl-lipid desaturase are desaturases that remove two hydrogen atoms from a fatty acid, creating a carbon/carbon double bond. They play an important role in PUFA synthesis. Long chain acyl-CoA synthetase can activate long chain and very long chain fatty acids to form acyl-CoAs. All of these genes are worthy of further investigation in future studies of PUFA synthesis.

Validation of DEGs by quantitative real-time PCR

To confirm the expression data from 454 pyrosequencing, quantitative real-time PCR (qRT-PCR) was performed to analyze the expression of candidate genes. Eleven up-regulated fatty acid

biosynthesis related genes in 20 DAF were selected for this verification, and 18S rRNA was used as an internal control. Only unigene3525 was not consistent with the sequencing results. The other 10 unigenes showed largely consistent results between qRT-PCR and 454 pyrosequencing (Figure 8).

Discussion

Oils extracted from plants have been widely used since ancient times in many countries. In addition, vegetable oils contain enhanced levels of health-promoting natural compounds and are associated with human health. However, researchers have found that a high intake of saturated and omega-6 fatty acids can increase the risk of cardiovascular disease (CVD) and cancer, in particular breast cancer, in recent years [2, 14]. At the same time, omega-3 PUFAs were shown to have chemopreventive properties against various cancers and their complications, including colon and breast cancer [15, 16]. These results suggest that a well-balanced omega-3/omega-6 fatty acid ratio will be beneficial for people’s health. Therefore, it is essential to increase the content of omega-3 fatty acids and reduce the omega-6 fatty acid contents in vegetable oils. Fish, such as salmon, herring, mackerel, anchovies and sardines, are a significant source of omega-3 long-chain PUFAs in the human diet [17]. With ocean exploitation increasing, reducing the amount of fish oil obtained from aquaculture is critical for sustainability and economic reasons [18]. A replacement for fish oil needs to be discovered urgently.

Much work has been done to engineer a sustainable land-based source of omega-3 long-chain PUFAs. Recently, the achievement of a high omega-3/omega-6 ratio through genetic and plant engineering was reported. The results indicated that both Arabidopsis and camelina transgenic

plants contained fish oil-like levels of DHA [9, 19]. Therefore, mining and characterization of PUFA biosynthesis genes are essential to improve the FA contents in plants by genetic engineering. In this study, our objective was to characterize the PUFA biosynthesis pathway genes active during seed development using 454 pyrosequencing. The expression levels of FA biosynthesis genes are induced before the early events of seed development [20, 21]. Our results showed that lipid content increased significantly from 10 to 25 DAF. Thus, 10 and 20 DAF samples were selected for expression profiling of camelina seeds. These results are in agreement with data published by Lee et al. [22] and Luo et al. [23].

By transcriptome sequence analysis, we obtained 831,632 clean reads, from which 32,759 predicted genes were subjected to BLAST annotation. The genome of *C. sativa* was sequenced recently and a total of 89,418 protein-coding genes were annotated [7]. This result confirmed the quality of our sequencing of camelina seeds. To investigate the PUFA biosynthesis pathway, we searched for fatty acid synthesis-associated genes across our sequencing results and found 220 up-regulated fatty acid biosynthesis genes in 20 DAF sample. Among them, several genes were characterized as key enzymes in FA biosynthesis (Figure 7). 3-Ketoacyl-acyl-carrier-protein reductase (FabG) was reported to be an essential enzyme for type II fatty acid biosynthesis and catalyzes an NADPH-dependent reduction of 3-ketoacyl-ACP to the (R)-3-hydroxyacyl isomer [24, 25]. Another key enzyme, enoyl-acyl-carrier-protein reductase (FabI), found in the FA biosynthesis pathway plays a determinant role in establishing the rate of FASII [26-28]. These results indicate that the genes shown in Figure 7 would play an important role in FA biosynthesis. Further studies are needed to determine the functions of these genes.

In a previous study, oleic acid (OA), LA and ALA were used as substrates for conversion to the

beneficial omega-3 long chain polyunsaturated fatty acid (LC-PUFA) EPA and DHA [9]. The content of unsaturated fatty acids in camelina is higher than in most other plants. In this study, we found 47 up-regulated PUFA biosynthesis-related genes in camelina seeds (Table 2). Twenty-one FAD genes were found and 13 of them were up-regulated and 6 were down-regulated (Additional file 5: Table S3). Ten up-regulated omega-6 FAD genes were found during seed development (Table 2). All of them were annotated as FAD2, which encodes an endoplasmic reticulum (ER) membrane-bound desaturase catalyzing conversion of OA to LA. Similarly, the expression levels of most *FAD2* genes were consistent with the results of Hutcheon et al. [5]. FAD2 was characterized to have a key role in the PUFA biosynthesis pathway in higher plant [29, 30]. LA account for about 93% omega-6 fatty acid (24.2% vs 25.9%) in camelina seeds [3], it will be mainly catalyzed by the omega-6 fatty acid desaturases. On the other hand, ALA makes up about 30% of the total fatty acid in camelina seeds [3]. Three FAD3 (unigene24351, 4386 and 23778) and three FAD7 (unigene13235, 17479 and 8495) were found in camelina transcriptome (Additional file 5: Table S3). However, only one FAD3 (unigene24351) was up-regulated during seed development. The expression level of unigene4386 and unigene13235 were induced slightly in 20 DAF sample. Unigene23778, unigene17479 and unigene8495 did not express in the 20 DAF sample, but they specifically expressed in 10 DAF sample. These results are consistently observed in the genome-wide analysis of *FAD3* in *Gossypium hirsutum*. The transcript level of *GhiFAD3-1* could be detected only in the early stage of *G. hirsutum* seed development [31]. In developing cotton fibers, the expression of *GhiFAD3-1* was down-regulated in both wild and domesticated *G. hirsutum* varieties [31]. These results suggest that ALA could be synthesized in the early stage of camelina and cotton developing seeds.

Other genes involved in PUFA biosynthesis were also found in this study, such as phosphatidylcholine diacylglycerol cholinephosphotransferase (PDAT) and acyl-CoA:diacylglycerol acyltransferase (DGAT). Triacylglycerol (TAG) can be formed via an acyl-CoA-dependent or acyl-CoA-independent process which catalyzed by PDAT and DGAT. The transcripts of 6 PDAT and 3 DGAT genes were found during camelina seed development stage (Table 2). All of them were up-regulated in 20 DAF sample. In previous study, overexpression of *Linum usitatissimum PDAT* and *DGAT* gene were characterized to produce more ALA in yeast strain H1246 [32, 33]. Moreover, overexpression of LuPDAT in Arabidopsis seed resulted in an enhanced level of PUFAs [32]. These results indicated that both PDAT and DGAT might have critical role in the TAG and PUFA biosynthesis in camelina seeds. Additionally, long chain acyl-CoA synthetases (ACSL) are key enzymes responsible for the conversion of acyl-AMP to acyl-CoA during fatty acid biosynthesis [34]. Here, we characterized 22 *ACSL* genes and 9 of them were up-regulated during seed development (Table 2). Therefore, the identified changes in gene expression in *C. sativa* may facilitate PUFA biosynthesis and the identification of related genes. This study will provide a resource for further studies on individual genes associated with fatty acid biosynthesis.

Conclusions

According to the pyrosequencing, 831,632 clean reads were obtained and 32,759 unigenes were predicted. All unigenes were analyzed with gene annotations from COG, KEGG, NR, NT and SwissProt databases. Among them, 220 up-regulated genes were identified as FA synthesis related genes (Additional files 4: Table S2), 47 of them are involved in PUFA biosynthesis (Table 2). Fifty-nine unigenes encoding *FAD2*, *FAD3*, *PDAT*, *DGAT* and *ACSL* genes were found in the

camelina transcriptome, most of them were up-regulated in the 20 DAF seeds. This transcriptome results provide a novel insight into the biosynthesis of polyunsaturated fatty acids. This research might represent a powerful tool to understand the molecular mechanisms of seed development and the result might be helpful for further gene expression, functional genomic studies and camelina molecular breeding.

Materials and Methods

Plant culture and collection

During 2011, eight rows (200 m row length and 50 cm spacing) of camelina were planted in the test plots of Jilin Agricultural University in Jilin Province, China at a uniform depth. The plants were subjected to irrigated and non-irrigated conditions until harvest. Irrigation was applied weekly to supplement recorded rainfall using above-ground drip irrigation as described by Campbell and Bauser [35]. The developmental processes of camelina seeds from flowering to seed maturity were observed from July to August 2011. Seeds were harvested at 10 DAF (immature stage), and then every 5 days until 40 DAF (mature stage). After removing the seed coat, the seeds were immediately frozen in liquid nitrogen for oil extraction and RNA isolation.

Measurement of oil content

To extract the oil (or lipids), seeds harvested at 10, 15, 20, 25, 30, 35 and 40 DAF were oven-dried at 85°C overnight. The dry samples were ground to a fine powder by a disintegrator, and the powder was transferred into glass tubes for oil extraction. Oil was extracted using ligarine to determine total lipids (TL) gravimetrically with the SER148 3/6 extraction apparatus (VELP Scientifica, Italy). Experiments were carried out using triplicate samples for each stage and mean

values were determined. Errors are shown as standard deviations. Statistical significance analyses were performed using *t*-test by SPSS (version 13.0, $P<0.05$).

Total RNA extraction and cDNA synthesis

Total RNA was extracted from these materials using TRIzol Reagent (Invitrogen, USA) following the manufacturer’s protocol. The quality of total RNA was determined using a NanoDrop Spectrometer (ND-1000 Spectrophotometer, Peqlab). The mRNAs were isolated from total RNAs using the PolyATtract mRNA Isolation Systems kit (Promega) and condensed using the RNeasy RNA cleaning kit (Qiagen, Germany); their concentration and purity were determined using the Agilent 2100 Bioanalyzer (RNA Nano Chip, Agilent). The mRNAs were fragmented and retrieved using an RNA Fragment reagent kit (Illumina) and RNeasy RNA cleaning kit (Qiagen). Then, random primers and M-MLV were used to synthesize the first chain, and DNA Polymerase I and RNase H were used to synthesize the second chain. Finally, the cDNAs were retrieved using the RNeasy RNA cleaning kit (Qiagen, Germany), and their quality was checked using the Agilent 2100 Bioanalyzer. All procedures were performed according to the manufacturers’ instructions.

454 sequencing and assembly

The raw 454 sequences in SFF files were base called using the python script sff_extract.py developed by COMAV (http://bioinf.comav.upv.es). All of the raw sequences were then processed to remove low quality and adaptor sequences using the programs tagdust [36], LUCY [37] and SeqClean [38] with default parameters. The resulting sequences were then screened against the NCBI UniVec database (http://www.ncbi.nlm.nih.gov/VecScreen/UniVec.html, version 20101122) to remove possible vector sequence contamination. Sequences shorter than 50 bp were discarded. The clean read sequences were assembled using MIRA3 [39] (minimum 30 bases

overlap with 80% identity) and CAP3 (overlap percent identity 90) [40]. The resulting contigs and singletons that were more than 100 nt long were retained as unigenes and annotated in the following steps.

Comparison analysis and functional annotation

To compare the differential expression of genes, we first recorded all reads of a unigene as the expression abundance. Then, expression data normalization was carried out using Reads Per Million reads (RPM) and Reads Per Kilo bases per Million reads (RPKM). The significance of differential gene expression was determined using the False Discovery Rate (FDR) and log2 ratio (T/C). Genes were deemed to be significantly differentially expressed with the threshold of "log2 ratio ≥ 1" and "FDR < 0.001" in sequence counts across the two samples.

Homolog searches against public sequence databases were performed to annotate the functions of the unigenes using BLAST with an E-value cutoff of 1e-6. The annotation of the record with highest similarity in the database was assigned as the functional annotation of the query unigene entry. The databases used for functional annotation included Nr (http://www.ncbi.nlm.nih.gov; version 20101011), Nt (http://www.ncbi.nlm.nih.gov, version 20101011) and SwissProt (http://www.ebi.ac.uk/uniprot, version 20090819). Additional functional classification was conducted using the COG (http://www.ncbi.nlm.nih.gov/COG/) and KEGG pathway (http://www.genome.jp/kegg) databases. ORF analysis was performed by ORF finder (http://www.ncbi.nlm.nih.gov/gorf/gorf.html).

Quantitative real-time PCR (qRT-PCR) analysis

Total RNA was extracted from seeds using TRIzol Reagent (Invitrogen) according to the manufacturer's protocol. cDNA was synthesized from 2 μg of total RNA using the PrimeScript RT

reagent Kit (Takara). Each reaction was performed in a 20 μL volume containing 10 μL SYBR Green Mastermix (Takara), 2 μL 50-fold diluted cDNA template and 1 μM each of the sense and anti-sense primers. qRT-PCR was performed on a Stratagene Mx3000P thermocycler (Agilent) with the following program: 95°C for 15 s, followed by 40 cycles of 95°C for 15s and annealing at 60°C for 30 s. Triplicates of each reaction were performed using *actin* as an internal reference. The gene-specific primers used for candidate genes are described in Additional file 7: Table S5.

Additional files

Additional file 1: Figure S1. Unsaturated fatty acid biosynthetic pathway in camelina. Red rectangles indicate up-regulated genes in 20 DAF sample. 3.1.2.2/TesB: Acyl-CoA thioesterase (Unigene8524).

Additional file 2: Figure S2. The quality analysis of mRNA and cDNA from *C. sativa* seeds. The mRNA and cDNA were examined by electrophoresis and shown in (A) and (B). The qualities of mRNA for the construction of cDNA library were further analyzed by Agilent2100 (C-F).

Additional file 3: Table S1. KEGG pathway annotation.

Additional file 4: Table S2. Up-regulated fatty acid biosynthesis genes in the 20 DAF sample.

Additional file 5: Table S3. Fatty acid desaturase genes involved in the PUFA synthesis pathway.

Additional file 6: Table S4. The number of matched proteins in different database.

Additional file 7: Table S5. Gene-specific primers used in qRT-PCR.

Availability of supporting data

The sequences used in this study have been submitted to the Sequence Read Archive at NCBI (Accession number: SRX866238).

Abbreviations

ALA, alpha linolenic acid; Ascl, long chain acyl-CoA synthetase; COG, cluster of orthologous groups of proteins; CVD, cardiovascular disease; DAF, days after flowering; DGAT, acyl-CoA:diacylglycerol acyltransferase; DEG, differentially expressed genes; ER, endoplasmic reticulum; FA, fatty acid; FabF, 3-oxoacyl-acyl-carrier-protein synthase; FabG, 3-ketoacy-acyl-carrier-protein reductase; FabI/FabK, enoyl-acyl-carrier-protein reductase; FAD, fatty acid desaturase; FAE, Fatty acid elongase; FDR, false disvovery rate; KEGG, Kyoto encyclopedia of genes and genomes; LA, linoleic acid; LC-PUFA, long chain polyunsaturated fatty acid; LPCAT, lysophosphatidylcholine acyltransferase; NADPH, nicotinamide adenine dinucleotide phosphate; OA, Oleic acid; PDAT, phospholipid:diacylglycerol acyltransferase; PDCT, phosphatidylcholine diacylglycerol cholinephosphotransferase; PUFA, polyunsaturated fatty acid; qRT-PCR, quantitative real time polymerase chain reaction; RPKM, reads per kilo bases per million reads; RPM, reads per million reads; SDA, stearidonic acid; TL, total lipids.

Competing Interests

The authors have declared that no competing interests exist.

Author contributions

Conceived and designed the experiments: FW, YD, HL. Performed the experiments: FW, HC, XL, JY, LG, NY, LD, YW, XL, XC. Analyzed the data: FW, YD, NW, ZW. Read and approved the final manuscript: FW, TW, HL.

Acknowledgements

This research was supported by the National “863” program (2011AA100606), the Special Program for Research of Transgenic Plants (2014ZX08010-002), the Development and Reform Commission of Jilin Province in China (JF2012C002-4), the National Natural Science Foundation of China (31271746, 31201144, 31101091, 31401403), and the Excellent Innovation Team Project of Jilin Province, China (20111815).

References

1. Deckelbaum RJ, Torrejon C (2012) The omega-3 fatty acid nutritional landscape: health benefits and sources. J Nutrition 142(3): 587S-591S.
2. De Lorgeril, Salen Patricia (2012) New insights into the health effects of dietary saturated and omega-6 and omega-3 polyunsaturated fatty acids. BMC Med 10: 50.
3. Hixson SM, Parrish CC, Anderson DM (2014) Changes in tissue lipid and fatty acid composition of farmed rainbow trout in response to dietary camelina oil as a replacement fo fish oil. Lipids 49(1): 97-111.
4. Eidhin DN, Burke J, O’Beirne D (2003) Oxidative stability of ω3-rich camelina oil and camelina oil-based spread compared with plant and fish oils and sunflower spread. J Food Sci 68(1): 345-353.
5. Hutcheon C, Ditt RF, Beilstein M, Comai L, Schroeder J, et al. (2010) Polyploid genome of *Camelina sativa* revealed by isolation of fatty acid synthesis genes. BMC Plant Biol 10: 233.
6. Kang JL, Snapp AR, Lu CF (2011) Identification of three genes encoding microsomal oleate desaturases (FAD2) from the oilseed crop *Camelina sativa*. Plant Physiol Biochem 49(2): 223-229.

7. Kagale S, Koh C, Nixon J, Bollina V, Clarke WE, et al. (2014) The emerging biofuel crop Camelina sativa retains a highly undifferentiated hexaploid genome structure. Nat Commun 23: 3706.
8. Sayanova O, Ruiz-Lopez N, Haslam RP, Napier JA (2012) The role of deta6-desaturase acyl-carrier specificity in the efficient synthesis of long-chain polyunsaturated fatty acids in transgenic plants. Plant Biotech J 10(2): 195-206.
9. Petrie JR, Shrestha P, Belide S, Kennedy Y, Lester G, et al. (2014) Metabolic engineering *Camelina sative* with fish oil-like levels of DHA. PLos One 9(1): e85061.
10. Mansour MP, Shrestha P, Belide S, Petrie JR, Nichols PD, et al. (2014) Characterization of oilseed lipids from "DHA-producing *Camelina sativa*": A new transformed land plant containing long-chain omega-3 oils. Nutrients 6(2): 776-789.
11. Nguyen HT, Silva JE, Podicheti R, Macrander J, Yang W, et al. (2013) Camelina seed transcriptome: a tool for meal and oil improvement and translational research. Plant Biotech J 11: 759-769.
12. Mudalkar S, Golla R, Ghatty S, Reddy AR (2014) De novo transcriptome analysis of an imminent biofuel crop, *Camelina sativa* L. using Illumina GAIIX sequencing platform and identification of SSR markers. Plant Mol Biol 84(1-2): 159-171.
13. Liang C, Liu X, Yiu SM, Lim BL (2013) De novo assembly and characterization of *Camelina sativa* transcriptome by paired-end sequencing. BMC Genomics 14: 146.
14. Siri-Tarno PW, Sun Q, Hu FB, Krauss RM (2010) Meta-analysis of prospective cohort studies evaluating the association of saturated fat with cardiovascular disease. Am J Clin Nutr 91(3): 535-546.

15. Cockbain AJ, Toogood GJ, Hull MA (2012) Oemga-3 polyunsaturated fatty acids for the treatment and prevention of colorectal cancer. Gut 61(1): 135-149.

16. Patterson RE, Flatt SW, Newman VA, Natarajan L, Rock CL, et al. (2011) Marine fatty acid intake is associated with breast cancer prognosis. J Nutr 141(2): 201-206.

17. Hixson SM, Parrish CC, Anderson DM (2013) Effect of replacement of fish oil with camelina (*Camelina sativa*) oil on growth, lipid class, and fatty acid composition of farmed juvenile Atlantic cod (*Gadus morhua*). Fish Physiol Biochem 39(6): 1441-1456.

18. Turchini G, Torstensen B, Wing-Keong N (2009) Fish oil replacement in finfish nutrition. Rev Aquac 1(1): 10-57.

19. Petrie JR, Shrestha P, Zhou XR, Mansour MP, Liu Q, et al. (2012) Metabolic engineering plant seeds with fish oil-like levels of DHA. PLOS One 7(11): e49165.

20. Chen H, Wang FW, Dong YY, Wang Nan, Sun YP, et al. (2012) Sequence mining and transcript profiling to explore differentially expressed genes associated with lipid biosynthesis during soybean seed development. BMC Plant Biol 12: 122.

21. Teoh KT, Requesens DV, Devaiah SP, Johnson D, Huang XZ, et al. (2013) Transcriptome analysis of embryo maturation in maize. BMC Plant Biol 13: 19.

22. Lee JM, Williams M, Tingey S, Rafalski A (2002) DNA array profiling of gene expression changes during maize embryo development. Funct Integr Genomics 2(1): 13-17.

23. Luo M, Liu J, Lee RD, Guo BZ (2008) Characterization of gene expression profiles in developing kernels of maize (Zea mays) inbred Tex6. Plant Breeding 127(6): 569-578.

24. Lai CY, Cronan JE (2004) Isolation and characterization of β-ketoacyl-acyl carrier protein reductase (*fabG*) mutants of *Escherichia coli* and *Salmonella enterica* serovar Typhimurium. J

Bacteriol 186: 1869-1878.

25. Tomura CT, Taguchi K, Gan Z, Kuwabara K, Tanaka T, et al. (2005) Expression of 3-ketoacyl-acyl carrier protein reductase (*fabG*) genes enhances production of polyhydroxyalkanoate copolymer from glycose in recombinant *Escherichia coli* JM109. Appl Environ Microbiol 71(8): 4297-4306.
26. Heath RJ, Rock CO (1995) Enoyl-acly carrier protein reductase (*fabI*) plays a determinant role in completing cycles of fatty acid elongation in *Escherichia coli*. J Biol Chem 270(44): 26538-26542.
27. Heath RJ, Rock CO (1996) Regulation of fatty acid elongation and initiation by acyl-acyl carrier protein in *Escherichia coli*. J Biol Chem 271(4): 1833-1836.
28. Yao JW, Abdelrahman YM, Robertson RM, Cox JV, Belland RJ, et al. (2014) Type II fatty acid synthesis is essential for the replication of *Chlamydia trachomatis*. J Biol Chem 289(32): 22365-22376.
29. Yadav NS, Wierzbicki A, Aegerter M, Caster CS, Perez-Grau L, et al. (1993) Cloning of higher plant ω-3 fatty acid desaturases. Plant Physiol 103: 467-476.
30. Chen JH, Zhu LH, Salentijn EM, Huang BQ, Gruber J, et al. (2013) Functional analysis of the omega-6 fatty acid desaturase (*CaFAD2*) gene family of the oil seed crop *Crambe abyssinica*. BMC Plant Biol 13: 146.
31. Yurchenko OP, Park S, Ilut DC, Inmon JJ, Millhollon JC, et al. (2014) Genome-wide analysis of the omega-3 fatty acid desaturase gene family in *Gossypium*. BMC Plant Biol 14: 312.
32. Pan X, Siloto RM, Wickramarathna AD, Mietkiewska E, Weselake RJ (2013) Identification of a pair of phospholipid:diacylglycerol acyltransferases from developing flax (*Linum*

usitatissimum L.) seed catalyzing the selective production of trilinolenin. J Biol Chem 288(33): 24173-24188.

33. Siloto RM, Truksa M, He X, McKeon T, Weselake RJ (2009) Simple methods to detect triacylglycerol biosynthesis in a yeast-based recombinant system. Lipids 44: 963-973.
34. Lopes-Marques M, Cunha I, Reis-Henriques MA, Santos MM, Castro LF (2013) Diversity and history of the long-chain acyl-CoA synthetase (*Acsl*) gene family in vertebrates. BMC Evol Biol 13: 271.
35. Campbell BT, Bauper PJ (2007) Genetic variation for yield and fiber quality response to supplemental irrigation within the Pee Dee Upland cotton germplasm collection. Crop Sci 47: 589-597.
36. Lassmann T, Hayashizaki Y, Daub CO (2009) TagDust—a program to eliminate artifacts from next generation sequencing data. Bioinformatics 25(21): 2839-2840.
37. Chen YA, Lin CC, Wang CD, Wu HB, Hwang PI (2007) An optimized procedure greatly improves EST vector contamination removal. BMC Genomics 8: 416.
38. Chevreux B, Wetter T, Suhai S (1999) Genome sequence assembly using trace signals and additional sequence information. Computer science and biology: Proceedings of the German conference on bioinformatics pp: 45-56.
39. Conesa A, Gotz S (2008) Blast2GO: A comprehensive suite for functional analysis in plant genomics. Int J Plant Genomics 2008: 619832.
40. Salomonis N, Hanspers K, Zambon AC, Vranizan K, Lawlor SC, et al. (2007) GenMAPP 2: new features and resources for pathway analysis. BMC Bioinformatics 8: 217.

Figure 1 Changes in lipid content during seed development. Lipid content was determined every 5 days. Values are means ± SE ($n = 3$). Significant difference compared with the control (10 DAF) is indicated with an asterisk ($P < 0.05$) .

Figure 2 Distribution of read lengths from the sequencing project.

Figure 3 Venn diagram of gene expression statistics in 10 and 20 DAF. The numbers 9081, 16317 and 7361 denote the 10 DAF-specific genes, overlapped genes, and 20 DAF-specific genes, respectively.

Figure 4 Analysis of differentially expressed genes in the two samples. A conventional log2 ratio threshold (≥ 1) was used to identify the DEGs.

Figure 5 COG function classification of all unigenes. The unigenes were classified into different functional groups based on COG annotations.

Figure 6 Distribution of multilevel COG annotation terms for the biological process category.

Figure 7 Fatty acid biosynthesis pathway in camelina. Red rectangles indicate up-regulated genes and green rectangles indicate down-regulated genes. FabF: 3-oxoacyl-acyl-carrier-protein synthase (Unigene2854, Unigene1012); FabG: 3-ketoacy-acyl-carrier-protein reductase (Unigene1548, Unigene22671 and Unigene11546); FabI/FabK: enoyl-acyl-carrier-protein reductase (Unigene28695, Unigene19796); 6.4.1.2/6.3.4.14: Acetyl-CoA carboxylase (Unigene18620, Unigene28036); 1.14.192: Acyl-ACP desaturase (Unigene 3928, Unigene29065, Unigene3732 and Unigene28370).

Figure 8 qRT-PCR validation of selected unigenes. The fold changes of the unigenes were calculated as the log2 ratio (20 DAF/10 DAF) for qRT-PCR. KPRM was selected to represent the

454 pyrosequencing results. Values are means ± SE with three replicates for each sample in qRT-PCR.

Table 1 Overview of sequencing, assembly and data statistics

	10 DAF	20 DAF
Raw reads	529324	318804
Low quality	1144	909
Short reads after primer clipped (<100bp)	32	6164
Contamination sequences	6465	1441
High repetitive	44	35
Vector sequences	132	130
Clean reads	521507	310125

Table 2 DEGs involved in the PUFA synthesis pathway

GeneID	Gene length	10 DAF Expression normalized	20 DAF Expression normalized	Fold(20 DAF/10 DAF)	log2 Ratio(20 DAF/10 DAF)	P-value
Unigene18620	142	0	106.89138	Inf	Inf	0.0000305
Unigene271	1003	0	65.577098	Inf	Inf	0
Unigene29085	266	0	19.020772	Inf	Inf	0.03125
Unigene7938	983	0	56.617272	Inf	Inf	0
Unigene28572	398	0	12.712375	Inf	Inf	0.03125
Unigene29065	385	0	13.141624	Inf	Inf	0.03125
Unigene3732	482	0	31.490821	Inf	Inf	0.0000305
Unigene18562	180	0	56.216948	Inf	Inf	0.0009766
Unigene24351	498	0	20.319379	Inf	Inf	0.0009766
Unigene27992	406	0	12.461885	Inf	Inf	0.03125
Unigene28768	510	0	9.9206379	Inf	Inf	0.03125
Unigene6131	649	0	15.591758	Inf	Inf	0.0009766
Unigene27436	333	0	15.19377	Inf	Inf	0.03125
Unigene28670	313	0	16.164618	Inf	Inf	0.03125
Unigene808	761	0	16	Inf	Inf	0
Unigene25348	100	0	101.19051	Inf	Inf	0.0009766
Unigene20594	693	0	14.601805	Inf	Inf	0.0009766
Unigene23255	547	0	18.499178	Inf	Inf	0.0009766
Unigene6196	878	0	28.812787	Inf	Inf	2.98E-08
Unigene25233	513	0	59.175735	Inf	Inf	9.31E-10
Unigene27635	447	0	11.318849	Inf	Inf	0.03125
Unigene28370	458	0	11.046999	Inf	Inf	0.03125
Unigene2120	866	0	58.42408	Inf	Inf	8.88E-16
Unigene27758	516	0	9.805282	Inf	Inf	0.03125
Unigene12780	484	0	62.72139	Inf	Inf	9.31E-10

Unigene2983	994	0	20.36026	Inf	Inf	9.54E-07
Unigene22028	539	0	18.77375	Inf	Inf	0.000977
Unigene21032	459	0	77.16052	Inf	Inf	2.91E-11
Unigene3928	844	4.8844234	317.71901	65.05	6.023419	4.59E-05
Unigene3902	1429	11.539408	354.06055	30.68	4.939355	4.01E-08
Unigene1155	1494	2.75934	57.57157	20.86	4.382962	4.59E-05
Unigene5146	610	6.7581203	66.35443	9.82	3.295499	4.59E-05
Unigene4015	985	8.3704637	77.048609	9.2	3.202389	5.24E-06
Unigene16451	450	9.1610075	67.460338	7.36	2.880461	4.94E-05
Unigene4010	1091	34.007406	245.78812	7.23	2.853494	1.86E-13
Unigene2346	363	22.713242	139.38086	6.14	2.617427	5.25E-06
Unigene529	746	16.578231	94.950877	5.73	2.517891	4.81E-07
Unigene1081	1953	16.88665	95.853782	5.68	2.504952	1.74E-12
Unigene2011	1777	16.23926	91.11132	5.61	2.488144	1.93E-11
Unigene238	1516	13.596482	63.410937	4.66	2.221498	3.18E-09
Unigene11605	550	14.99074	55.19482	3.68	1.880461	0.000299
Unigene17237	439	9.3905543	34.575344	3.68	1.880461	0.0118639
Unigene7439	1526	16.20886	46.417663	2.86	1.517891	1.46E-06
Unigene3885	1584	309.70451	798.53619	2.58	1.366465	0
Unigene21006	843	2	4	2.45	1.295499	0.024125
Unigene4022	1103	11.212475	22.935292	2.05	1.032464	0.033553
Unigene8095	923	26.79818	54.816092	2.05	1.032464	0.0024442

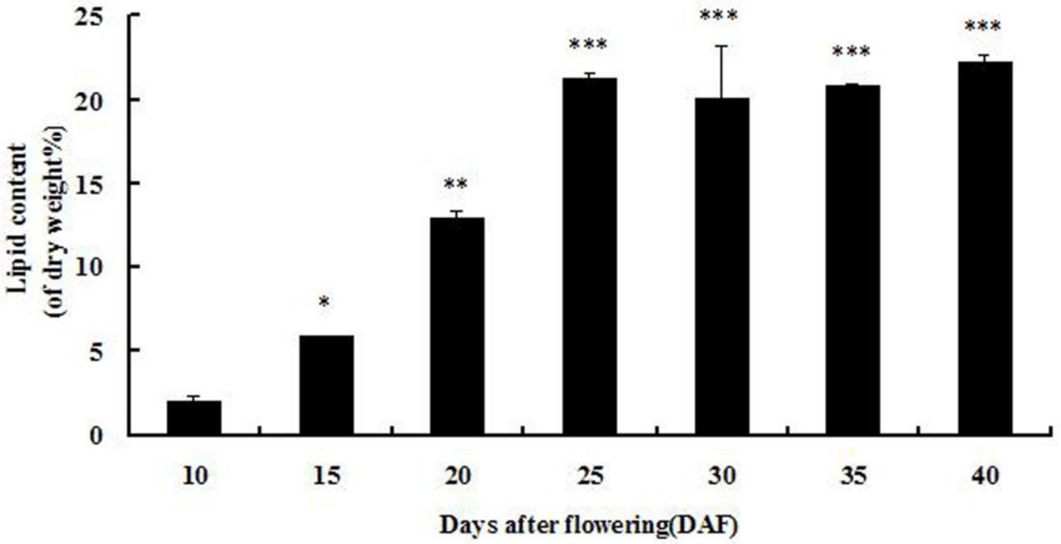

Lipid content
(of dry weight%)
25
20
15
10
5
0
*
**
***
***
***
***
10
15
20
25
30
35
40
Days after flowering(DAF)

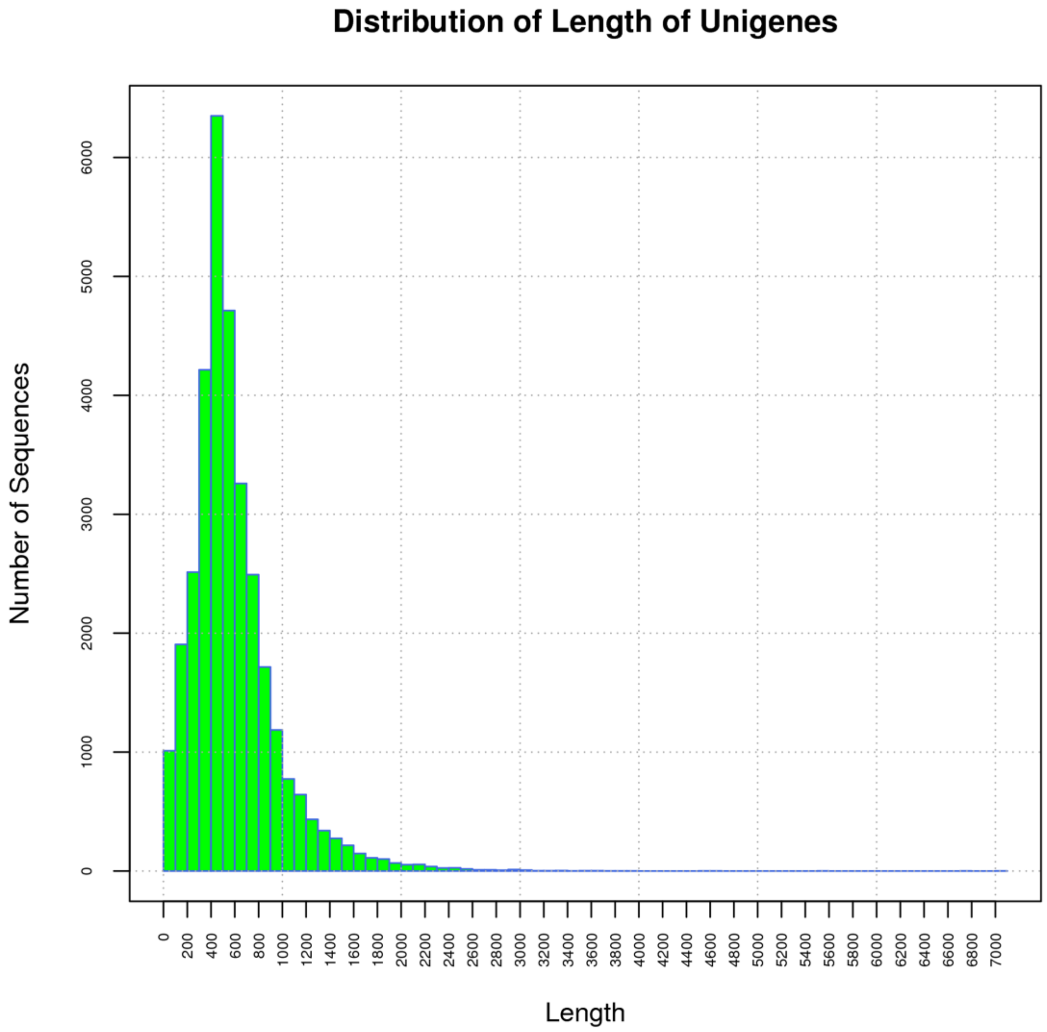
Distribution of Length of Unigenes
Number of Sequences
Length
0
1000
2000
3000
4000
5000
6000
0
200
400
600
800
1000
1200
1400
1600
1800
2000
2200
2400
2600
2800
3000
3200
3400
3600
3800
4000
4200
4400
4600
4800
5000
5200
5400
5600
5800
6000
6200
6400
6600
6800
7000

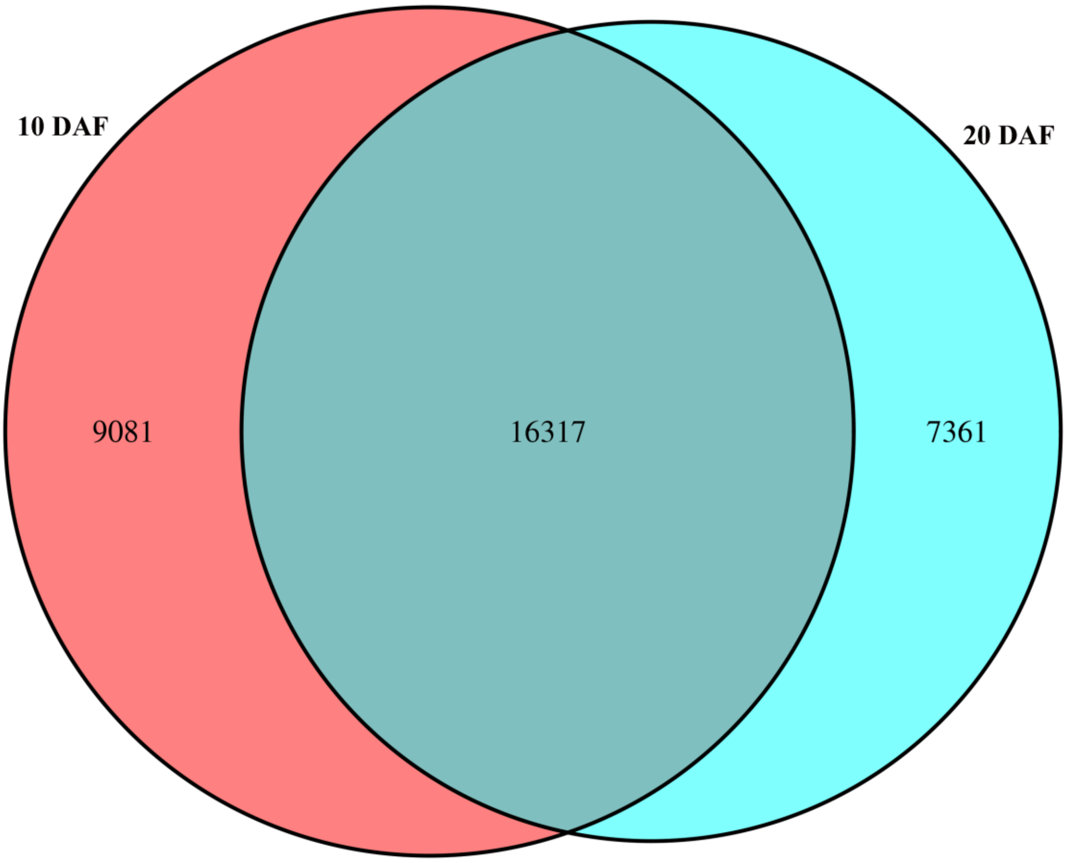
10 DAF
20 DAF
9081
16317
7361

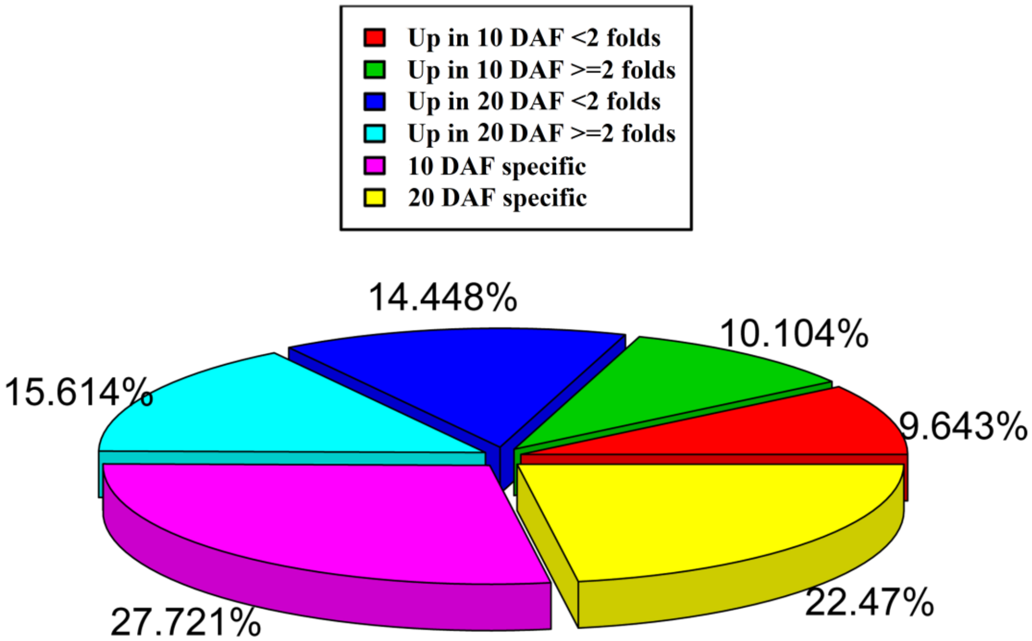
Up in 10 DAF <2 folds
Up in 10 DAF >=2 folds
Up in 20 DAF <2 folds
Up in 20 DAF >=2 folds
10 DAF specific
20 DAF specific
14.448%
10.104%
15.614%
9.643%
22.47%
27.721%

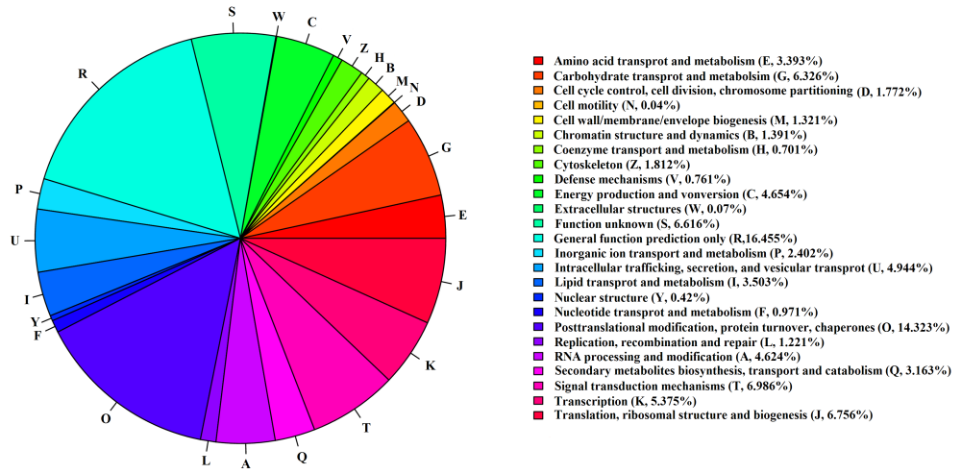
S
W
C
V
Z
H
B
M
N
D
G
E
J
K
T
Q
A
L
O
F
Y
I
U
P
R
Amino acid transprot and metabolism (E, 3.393%)
Carbohydrate transprot and metabolsim (G, 6.326%)
Cell cycle control, cell division, chromosome partitioning (D, 1.772%)
Cell motility (N, 0.04%)
Cell wall/membrane/envelope biogenesis (M, 1.321%)
Chromatin structure and dynamics (B, 1.391%)
Coenzyme transport and metabolism (H, 0.701%)
Cytoskeleton (Z, 1.812%)
Defense mechanisms (V, 0.761%)
Energy production and vonversion (C, 4.654%)
Extracellular structures (W, 0.07%)
Function unknown (S, 6.616%)
General function prediction only (R,16.455%)
Inorganic ion transport and metabolism (P, 2.402%)
Intracellular trafficking, secretion, and vesicular transprot (U, 4.944%)
Lipid transprot and metabolism (I, 3.503%)
Nuclear structure (Y, 0.42%)
Nucleotide transprot and metabolism (F, 0.971%)
Posttranslational modification, protein turnover, chaperones (O, 14.323%)
Replication, recombination and repair (L, 1.221%)
RNA processing and modification (A, 4.624%)
Secondary metabolites biosynthesis, transport and catabolism (Q, 3.163%)
Signal transduction mechanisms (T, 6.986%)
Transcription (K, 5.375%)
Translation, ribosomal structure and biogenesis (J, 6.756%)

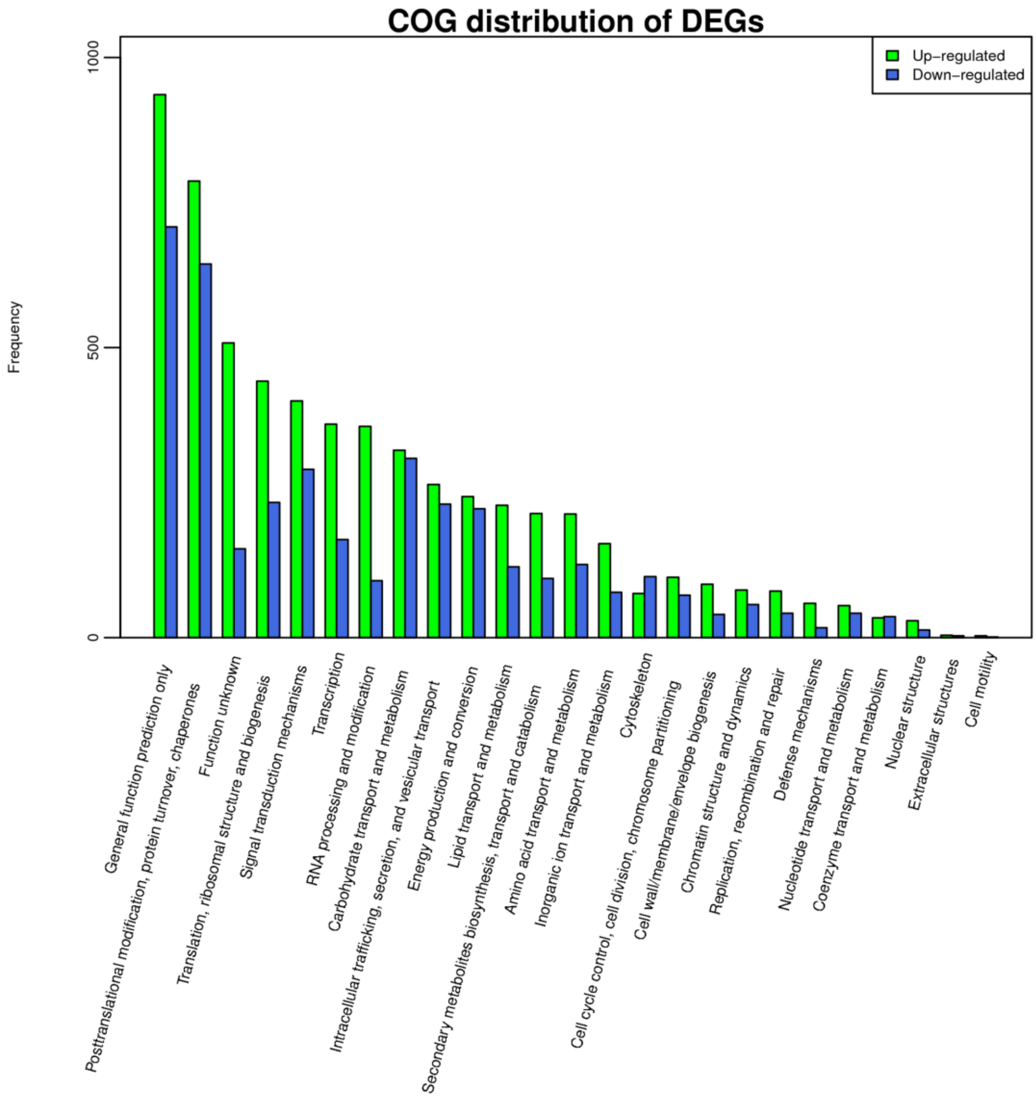
COG distribution of DEGs
Up−regulated
Down−regulated
Frequency
1000
500
0
General function prediction only
Posttranslational modification, protein turnover, chaperones
Function unknown
Translation, ribosomal structure and biogenesis
Signal transduction mechanisms
Transcription
RNA processing and modification
Carbohydrate transport and metabolism
Intracellular trafficking, secretion, and vesicular transport
Energy production and conversion
Lipid transport and metabolism
Secondary metabolites biosynthesis, transport and catabolism
Amino acid transport and metabolism
Inorganic ion transport and metabolism
Cytoskeleton
Cell cycle control, cell division, chromosome partitioning
Cell wall/membrane/envelope biogenesis
Chromatin structure and dynamics
Replication, recombination and repair
Defense mechanisms
Nucleotide transport and metabolism
Coenzyme transport and metabolism
Nuclear structure
Extracellular structures
Cell motility

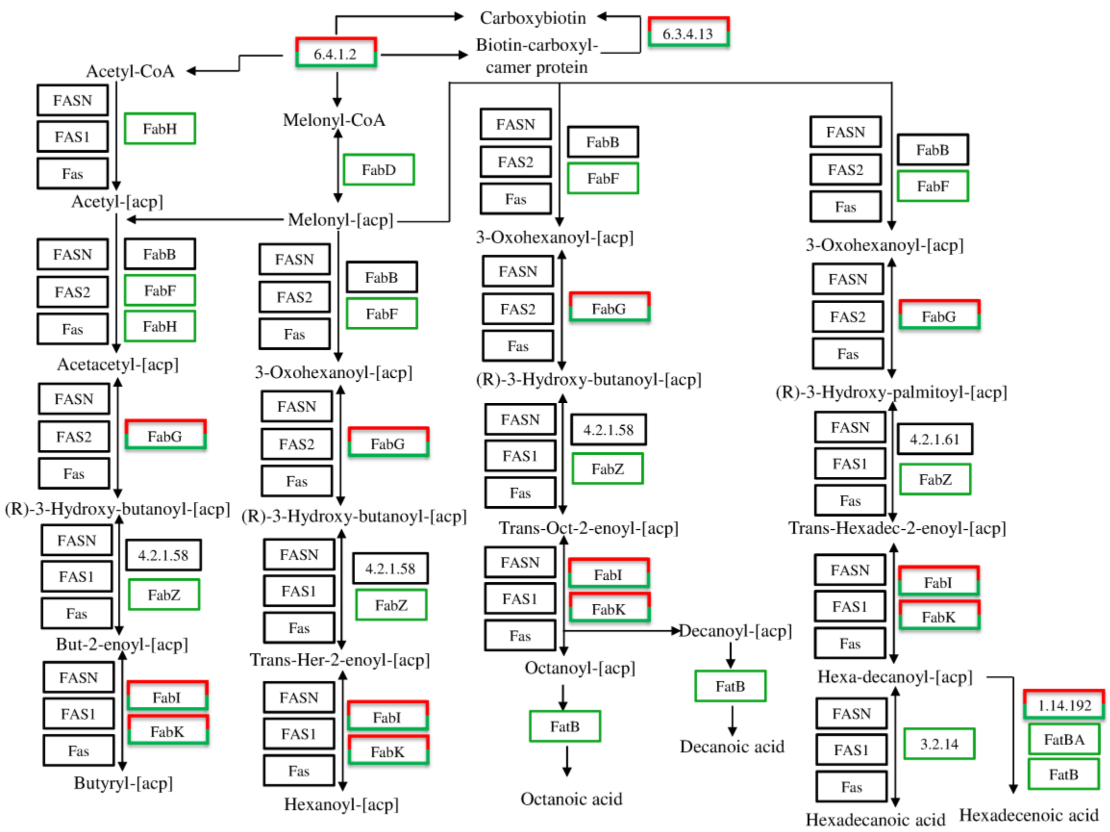

Carboxybiotin
6.3.4.13
6.4.1.2
Biotin-carboxyl-camer protein
Acetyl-CoA
Melonyl-CoA
FabD
Melonyl-[acp]
FASN
FAS1
Fas
FabH
Acetyl-[acp]
FASN
FAS2
Fas
FabB
FabF
FabH
Acetacetyl-[acp]
FabG
(R)-3-Hydroxy-butanoyl-[acp]
4.2.1.58
FabZ
But-2-enoyl-[acp]
FabI
FabK
Butyryl-[acp]
3-Oxohexanoyl-[acp]
(R)-3-Hydroxy-butanoyl-[acp]
Trans-Her-2-enoyl-[acp]
Hexanoyl-[acp]
3-Oxohexanoyl-[acp]
(R)-3-Hydroxy-butanoyl-[acp]
Trans-Oct-2-enoyl-[acp]
Decanoyl-[acp]
Octanoyl-[acp]
FatB
Octanoic acid
Decanoic acid
3-Oxohexanoyl-[acp]
(R)-3-Hydroxy-palmitoyl-[acp]
4.2.1.61
Trans-Hexadec-2-enoyl-[acp]
Hexa-decanoyl-[acp]
3.2.14
1.14.192
FatBA
Hexadecanoic acid
Hexadecenoic acid

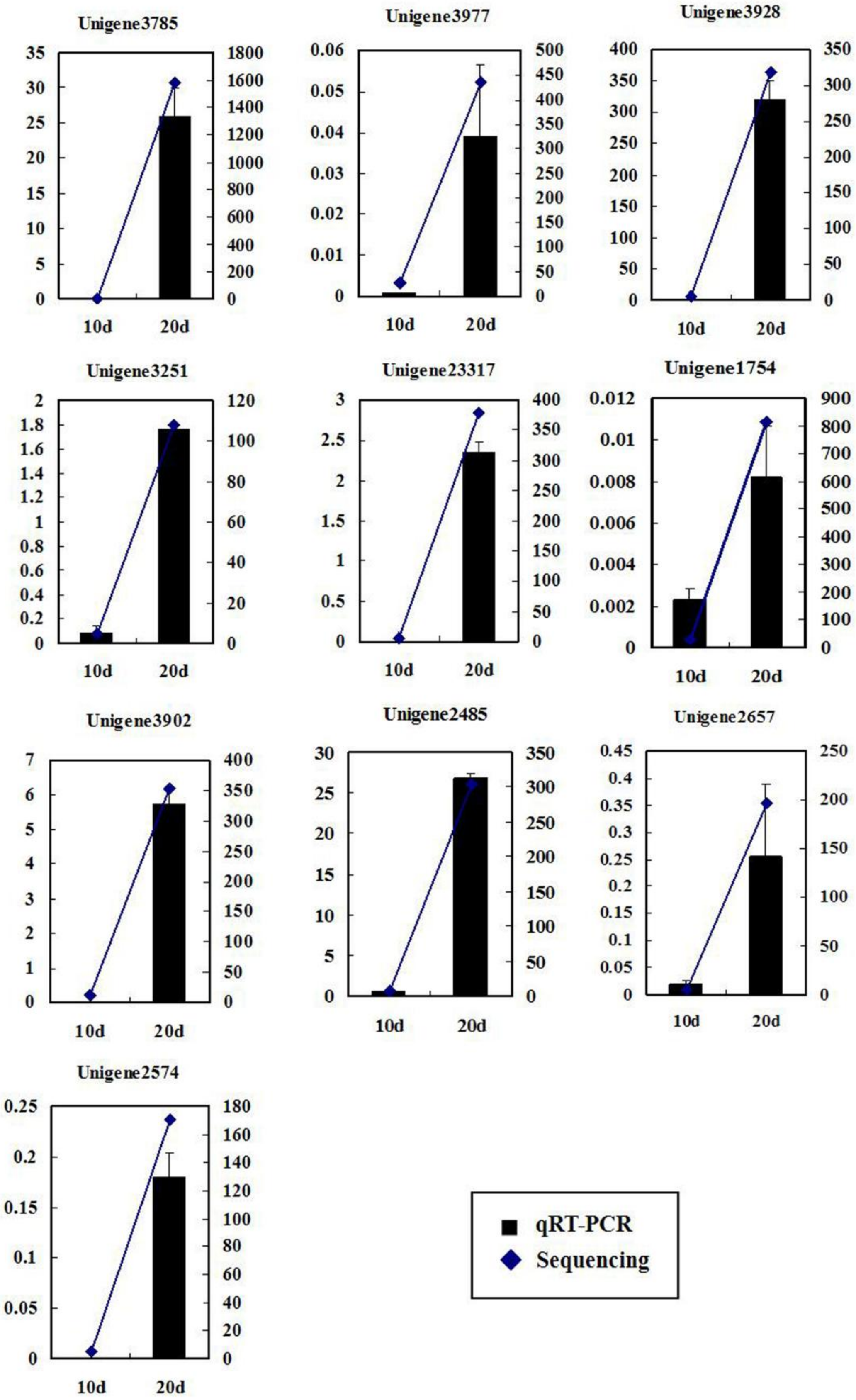

Unigene3785
35 30 25 20 15 10 5 0
1800 1600 1400 1200 1000 800 600 400 200 0
10d 20d
Unigene3977
0.06 0.05 0.04 0.03 0.02 0.01 0
500 450 400 350 300 250 200 150 100 50 0
10d 20d
Unigene3928
400 350 300 250 200 150 100 50 0
350 300 250 200 150 100 50 0
10d 20d
Unigene3251
2 1.8 1.6 1.4 1.2 1 0.8 0.6 0.4 0.2 0
120 100 80 60 40 20 0
10d 20d
Unigene23317
3 2.5 2 1.5 1 0.5 0
400 350 300 250 200 150 100 50 0
10d 20d
Unigene1754
0.012 0.01 0.008 0.006 0.004 0.002 0
900 800 700 600 500 400 300 200 100 0
10d 20d
Unigene3902
7 6 5 4 3 2 1 0
400 350 300 250 200 150 100 50 0
10d 20d
Unigene2485
30 25 20 15 10 5 0
350 300 250 200 150 100 50 0
10d 20d
Unigene2657
0.45 0.4 0.35 0.3 0.25 0.2 0.15 0.1 0.05 0
250 200 150 100 50 0
10d 20d
Unigene2574
0.25 0.2 0.15 0.1 0.05 0
180 160 140 120 100 80 60 40 20 0
10d 20d
qRT-PCR
Sequencing
